# Genomic characterization and comparative analysis of multidrug-resistant *Salmonella* Indiana sequence type 17 carrying *mcr-9.1* in retail eggs from the United Arab Emirates

**DOI:** 10.3389/fvets.2026.1825641

**Published:** 2026-06-09

**Authors:** Ihab Habib, Fatma A. Mohamed, Mohammed Elbediwi, Akela Ghazawi, Glindya Bhagya Lakshmi, Xander Velkeneers, Loïc Lesobre, Ana Perez de Vargas, Mushtaq Khan, Rami H. Al-Rifai

**Affiliations:** 1 Veterinary Public Health Research Laboratory, Department of Veterinary Medicine, College of Agriculture and Veterinary Medicine, United Arab Emirates University, Al Ain, United Arab Emirates; 2 ASPIRE Research Institute for Food Security in the Drylands (ARIFSID), United Arab Emirates University, Al Ain, United Arab Emirates; 3 Department of Microbiology and Immunology, Faculty of Pharmacy, Zagazig University, Zagazig, Egypt; 4 Department of Medical Microbiology and Immunology, University of Pécs Medical School, Pécs, Hungary; 5 University Institute for Medical Microbiology and Virology, Carl von Ossietzky University Oldenburg, Oldenburg, Germany; 6 Animal Health Research Institute, Agriculture Research Centre, Cairo, Egypt; 7 Department of Medical Microbiology and Immunology, College of Medicine and Health Sciences, United Arab Emirates University, Al Ain, United Arab Emirates; 8 OIKOS Genomics, Abu Dhabi, United Arab Emirates; 9 Zayed Bin Sultan Center for Health Sciences, United Arab Emirates University, Al Ain, United Arab Emirates; 10 Infectious Diseases Epidemiology Research Advancement Unit (IDERA), Institute of Public Health, College of Medicine and Health Sciences, United Arab Emirates University, Al Ain, United Arab Emirates

**Keywords:** colistin, eggs, foodborne antimicrobial resistance, salmonella, United Arab Emirates, whole genome sequencing

## Abstract

**Introduction:**

The occurrence and genomic context of the mobile colistin resistance gene *mcr-9.1* in the United Arab Emirates (UAE) food chain remain poorly characterized, with no previous reports identified from retail food products based on the available literature and public genomic databases. *Salmonella enterica* is a leading foodborne zoonotic pathogen within the One Health framework. This study reports, to the best of our knowledge, the first detection of the *mcr-9.1* gene in the UAE, identified during a baseline survey of *Salmonella* in retail table eggs.

**Methods:**

A total of 380 consumer egg packs were collected from supermarkets in the emirates of Abu Dhabi and Dubai between November 2024 and January 2025. *Salmonella* isolation followed ISO 6579-1:2017 protocols, with confirmed isolates underwent antimicrobial susceptibility testing and whole-genome sequencing using Illumina and Oxford Nanopore platforms. Hybrid assemblies were generated and analyzed using genomics workflow for antimicrobial resistance (AMRFinderPlus), plasmid typing (MOB-suite), virulence profiling (VirulenceFinder), and phylogenetic placement (kSNP4, FastTree 2.1.10).

**Results and discussion:**

Out of 380 egg samples tested, 37 (9.7%; 95% CI 7.2%−13.1%) were *Salmonella*-positive. Of which, two (5.4%) *S*. Indiana isolates (F-9 and F-32), both sequence type ST17, were identified to carry *mcr-9.1* yet remained phenotypically susceptible to colistin (MIC ≤ 1 mg/L). These isolates displayed multidrug resistance, harboring aminoglycoside, tetracycline, sulfonamide, and quinolone resistance genes alongside *mcr-9.1*. Hybrid genome assembly localized *mcr-9.1* on a non-replicon-bearing large contig likely associated with the chromosome, with an adjacent *IS3*-family element (*ISSen1*) suggesting potential mobility. Phylogenetic analysis positioned both isolates within a clade of Asian poultry- and food-origin *S*. Indiana ST17, differing by only six SNPs from each other. To the best of our knowledge, this study documents the first detection of *mcr-9.1*-positive *Salmonella* in the UAE, highlighting its putative chromosomal integration in multidrug-resistant *S*. Indiana ST17 isolates from retail eggs. These findings support targeted AMR surveillance of retail egg products and food-associated *Salmonella* in the UAE, including routine screening for *mcr* genes and WGS of multidrug-resistant isolates. Linking genomic data with product origin and distribution metadata would improve the ability to monitor the emergence, persistence, and possible dissemination routes of colistin resistance determinants in the food chain.

## Introduction

Zoonotic salmonellosis remains a major global veterinary public health concern (1). Each year roughly 93.8 million cases of enteric illness and 150,000 deaths are attributed to *Salmonella* infection (1). Human infection is closely linked to animal-source foods, with contaminated poultry meat and shell eggs being among the principal vehicles of transmission (2, 3). Over the past two decades, the emergence and dissemination of antimicrobial resistance (AMR) among non-typhoidal *Salmonella* (NTS) have added a significant layer of complexity to public health and food safety challenges (4). The World Health Organization has categorized fluoroquinolone-resistant *Salmonella* as a high-priority pathogen for research and development of new antibiotics (4). Added to that, the spread of resistance to colistin among NTS is particularly concerning because colistin is a last resort polymyxin used to treat infections caused by multidrug-resistant Gram-negative bacteria (5, 6).

Colistin resistance was once thought to arise solely through chromosomal mutations, but the discovery of plasmid-mediated Mobile Colistin Resistance (*mcr*) genes in 2015 marked a paradigm shift (7). Ten distinct *mcr* variants (*mcr-1* to *mcr*-*10*) have since been identified in Enterobacterales across all continents (7). Among the *mcr* variants, *mcr-9* has attracted particular attention in recent years. First detected in 2019 through *in silico* analysis of archived *Salmonella* genomes, *mcr*-*9* has since been identified in multiple NTS serovars across diverse One Health environments (8). Unlike *mcr-1, mcr-9* does not always confer phenotypic resistance; its expression can be silent or inducible, enabling the gene to persist undetected in bacterial populations (9). The silent spread of certain *mcr* genes undermines the efficacy of one of the few remaining therapies for carbapenem-resistant infections and represents a pressing One Health challenge (6). Understanding the dynamics of *mcr-9* carriage is therefore essential to protect the efficacy of polymyxins and other antibiotics.

Emerging *Salmonella* serovars further complicate the epidemiological landscape by introducing additional genetic variability and transmission dynamics across different hosts and environments (10). *Salmonella enterica* subsp. *enterica* serovar Indiana (hereafter will be addressed as *S*. Indiana) is a high-risk serovar that has been increasingly isolated from poultry and humans particularly in Asia in recent years (11, 12). It is often harboring multidrug-resistant determinants and occasionally *mcr* genes (11, 12). Studies from China have reported *S*. Indiana carrying *mcr-1* on IncHI2 or IncX4 plasmids but reports of *mcr-9* in foodborne isolates among this serovar are rare (12, 13).

The United Arab Emirates (UAE) has experienced rapid growth in poultry production and consumption, yet published data on AMR in this sector is limited. Recent surveillance has uncovered plasmid-borne *mcr* genes in diverse settings. In locally produced chicken meat, 12 colistin-resistant *Escherichia coli* isolates carrying *mcr-1.1* on IncI2 plasmids were reported (14), and a baseline survey of chilled chicken carcasses recovered *Salmonella* Minnesota with *mcr-1.1* (15). In 2025, researchers documented the first *mcr-1.1*-positive *Salmonella* Infantis isolates from a broiler farm in the UAE (16), emphasizing co-carriage of multiple resistance and the potential for cross-sector dissemination. However, to the best of our knowledge, *mcr-9* has not been reported in the UAE from any source, including clinical specimens, environmental reservoirs, or foodborne isolates, suggesting that its occurrence may remain relatively limited within local microbial populations.

Here we present what appears to be the first detection of the mobile colistin resistance gene *mcr-9.1* in the UAE retail egg supply chain, based on our review of the available literature and public genomic databases, within the context of a baseline study (17). By employing whole-genome sequencing, we aimed to characterize the genetic features of the recovered *mcr-9.1*-harboring isolates and assess their relevance to AMR surveillance in the retail food chain, while acknowledging their potential relevance to broader One Health-oriented surveillance efforts. Epidemiological analyses arising from the baseline survey of *Salmonella* in retail eggs in the UAE, including prevalence and distribution patterns, are outside the scope of this manuscript and have been reported separately (17).

## Materials and methods

### Survey context and isolations

The strains described in this study were obtained from a baseline survey investigating *Salmonella* contamination in retail eggs from supermarkets across the UAE (17). A total of 380 consumer egg packs (12 eggs per pack) were analyzed for *Salmonella* detection. Each egg was aseptically cracked open using a sterile scalpel blade (Swann-Morton, UK) at the blunt end to minimize shell contamination. The egg contents of each pack were poured into a sterile stomacher bag (Seward, UK). Eggshells (pool of 12 per sample unit) were rinsed with sterile phosphate-buffered saline (PBS; Oxoid, UK) to remove residual albumen, pooled, and crushed manually in sterile stomacher bags triple-bagged to prevent leakage (17, 18).

Isolation of *Salmonella* followed ISO 6579-1:2017, where eggshells and egg contents were handled separately throughout the pre-enrichment and enrichment steps (18). Briefly, the crushed shells were weighed, and buffered peptone water (BPW; Oxoid, UK) was added at a 1:9 ratio. Homogenization was performed in a stomacher (Seward, UK) for 1 min, followed by incubation at 37 °C for 48 h. After pre-enrichment, 1 ml of homogenate was inoculated into Mueller-Kauffmann Tetrathionate Novobiocin broth (MKTTn; Oxoid, UK), and 0.1 ml was spotted onto Modified Semi-solid Rappaport-Vassiliadis medium (MSRV; Oxoid, UK). MKTTn broths were incubated at 37 °C for 24 h, and MSRV plates at 41.5 °C for 24–48 h, examining for typical migration zones (17, 18). From both enrichment media, aliquots were streaked on Xylose Lysine Deoxycholate (XLD; Oxoid, UK) and incubated at 37 °C for 24 h. Up to five presumptive *Salmonella* colonies were sub-cultured onto Nutrient Agar (Oxoid, UK) and incubated for 24 h at 37 °C (17, 18). Isolates were confirmed to species level using Matrix-Assisted Laser Desorption Ionization-Time of Flight Mass Spectrometry (MALDI-TOF MS; Autobio Diagnostics, China).

### Sequencing and genome assembly

Genomic DNA was extracted using the DNeasy Blood and Tissue Kit (Qiagen, Hilden, Germany) according to the manufacturer's instructions. DNA quantity and purity were assessed using a NanoDrop™ spectrophotometer (Thermo Fisher Scientific, Waltham, MA, USA) and a Quantus™ Fluorometer (Promega, Madison, WI, USA). DNA samples were then shipped to Novogene Europe Genomics (Cambridge, United Kingdom) for Illumina short-read sequencing, using the Illumina DNA Prep Kit (Illumina Inc., San Diego, CA, USA) and sequenced on a NovaSeq platform (15). The short-read sequencing pointed out that among the confirmed *Salmonella*-positive samples, two *S*. Indiana isolates (F-9 and F-32) harbored *mcr-9.1 gene* (Table 1). Both isolates originated from table-egg samples of the same retail brand (same producer), collected independently from supermarkets in Dubai (November 2024) and Al Ain (January 2025) (Table 1). For these two isolates, Oxford Nanopore long-read sequencing was performed by OIKOS Genomics (Abu Dhabi, UAE), where libraries were prepared with the Rapid Barcoding Kit (SQK-RBK114.24), and the Rapid Barcoding Kit (SQK-RBK114.96). Libraries were sequenced on a P2 Solo device equipped with a PromethION flow cell (FLO-PRO114M; Oxford Nanopore Technologies, Oxford, UK) (19).

**Table 1:** Survey context and characteristics of the two colistin-susceptible *mcr-9.1*-positive *Salmonella* Indiana isolated from retail table egg samples in the United Arab Emirates.

The context for the survey	- Retail table egg samples (***n*** = 380) were collected from supermarkets across Abu Dhabi and Dubai Emirates. ***- Salmonella*** prevalence was 9.7 % ((37/380); 95 % CI 7.2%−13.1%), and whole-genome sequencing was achieved for all recovered isolates (17).	
Colistin minimum inhibitory concentration (MIC)	≤ 1 mg/L (wildtype/susceptible)	≤ 1 mg/L (wild type/susceptible)
15.6-7.8,-26.0498.8ptAntimicrobial resistance profile (MIC)	Gentamicin (8 mg/L), ciprofloxacin (0.12 mg/L), trimethoprim/sulfamethoxazole (>512 mg/L), tetracycline (>32 mg/L).	Gentamicin (4 mg/L), ciprofloxacin (0.25 mg/L), nalidixic acid (16 mg/L), trimethoprim/sulfamethoxazole (>512 mg/L), tetracycline (>32 mg/L).
WGS characterization		
Predicted serotype	Indiana (isolate's code: F-9)	Indiana (isolate's code: F-32)
Predicted antigenic profile	4:z:1,7	4:z:1,7
Sequence type	17	17
Antimicrobial resistance genes	*mdsB, mdsA, ant(2”)-Ia, aadA2, sul1, qnrA1, aph(3')-Ia, tet(D), mcr-9.1*	*mdsB, mdsA, ant(2‘')-Ia, aadA2, sul1* *qnrA1, aph(3')-Ia, tet(D), mcr-9.1*
Stress resistance genes	*golT, golS, qacEdelta1, qacE, merE, merD merA, merT, merR, arsC, pcoS*	*golT, golS, qacEdelta1, arsC, pcoS, merE* *merD, merA, merT, merR*
Predicted plasmids	MOB Cluster AA400: - 63661 bp - Non-mobilizable - Resistance genes: *aadA2, ant(2”)-Ia, aph(3')-Ia, qacE, qacEdelta1, qnrA1, sul1, tet(D)*	MOB Cluster AA400: - 62749 bp - Non-mobilizable - Resistance genes: *aadA2, ant(2”)-Ia, aph(3')-Ia, qacEdelta1, qnrA1, sul1, tet(D)*
	MOB Cluster AF643: - 23525 bp - Non-mobilizable - Resistance genes: none.	
Assembly quality statistics	- No. of Illumina contigs: 52 - No. of ONT contigs: 28 - N50 (Illumina): 189,937 - N50 (ONT): 409,691	- No. of Illumina contigs: 91 - No. of ONT contigs: 30 - N50 (Illumina): 120,911 - N50 (ONT): 230,684
Total length	4.72 Mbp	4.68 Mbp
BioSample number	SAMN50985254	SAMN50985277
Origin	Local (UAE-based producer) Supermarket in Dubai city (November 2024)	Local (UAE-based producer) Supermarket in Al Ain city (January 2025)

A hybrid genome assembly of the *S*. Indiana F-9 and F-32 strains was performed using Unicycler v0.5.1 in *bold* mode, which includes overlap identification, trimming, and rotation of the final assembly to start at the *dnaA/repA* gene (19). The genomic sequencing data and assemblies for strains *S*. Indiana F-9 and F-32 have been deposited in the NCBI database under BioProject number PRJNA1314772 (https://www.ncbi.nlm.nih.gov/bioproject/1314772). The corresponding BioSample numbers are SAMN50985254 (*S*. Indiana F-9) and SAMN50985277 (*S*. Indiana F-32).

### Genome analysis and phylogenetic reconstruction

For genomic characterization, FASTQ files from the genomes of *S*. Indiana strains (F-9 and F-32) were uploaded to the Solu Genomics cloud-based bacterial genomics platform (https://www.solugenomics.com). The Solu Genomics workflow, described in detail elsewhere (20), integrates a suite of validated bioinformatics pipelines for bacterial genome assembly, typing, resistome, plasmid, and virulence traits analysis. Briefly, species confirmation was conducted using Kraken2 v2.1.2 with abundance re-estimation by Bracken, while serovar prediction was performed using SISTR v1.1.1. Multilocus sequence typing (MLST) was assigned using mlst v2.23.0 with the *Salmonella* PubMLST scheme (20). The AMR determinants and chromosomal point mutations were identified using AMRFinderPlus v3.11, applying its default thresholds (≥90% sequence identity and ≥50% coverage for acquired genes, with curated criteria for point mutations). Plasmid replicon types were detected using MOB-suite v3.1.0 with default parameters, which rely on reference-based clustering without fixed identity cutoffs. Virulence-associated loci were screened using VirulenceFinder v2.0, using the default thresholds of ≥90% nucleotide identity and ≥60% minimum gene coverage; all of the former mentioned pipelines were implemented through the Solu Genomics cloud-based platform (20).

To investigate the phylogenetic relatedness and potential One Health transmission links of *S*. Indiana sequence type 17 (ST17) isolates, core genome single nucleotide polymorphism (cgSNP) analysis was performed. Genomes from the two strains characterized in this study (F-9 and F-32) were compared with 155 publicly available ST17 *Salmonella* genomes from Asian countries retrieved from the EnteroBase database (accessed May 2025). The aim of this comparison was to place the UAE isolates within the broader regional epidemiological context, given the country's geographic position in West Asia and its potential role in linking *Salmonella* ST17 lineages circulating across Asian regions (11, 12). The metadata for those 155 isolates are provided in Supplementary Table S1 (with 35 of them were found to be missing some of the identification data, mainly the year of isolation). SNP identification was conducted using kSNP4 v4.0 (21), a reference-free, *k*-mer–based pipeline for detecting high-resolution SNP differences across bacterial genomes. A maximum likelihood (ML) phylogenetic tree was inferred using FastTree 2.1.10 (22). The resulting tree was visualized using iTOL (interactive tree of life) v7 (https://itol.embl.de/) to incorporate metadata related to the isolates' source (human, food, animal, or environment within the Asian region) to reflect the One Health context of any potential regional transmission dynamics.

The genetic environment surrounding the *mcr-9.1* gene in two *S*. Indiana isolates (F-32 and F-9) was analyzed using the Easyfig v2.2.5 pipeline (http://mjsull.github.io/Easyfig/). Genomic regions encompassing *mcr-9.1* and adjacent open reading frames were extracted from assembled genomes and annotated based on GenBank annotations. Comparative analyses were performed against representative chromosomal and plasmid sequences retrieved from the NCBI database, including complete chromosome and plasmid accessions identified by BLASTn searches (https://blast.ncbi.nlm.nih.gov/). Easyfig was used to visualize gene synteny, orientation, and homology across the selected sequences; and reference sequences were grouped according to chromosomal or plasmid origin to facilitate comparison of *mcr-9.1* genomic contexts across different bacterial hosts.

We checked the available published literature (PubMed search) and public sequence databases for previous reports of *mcr-9*/*mcr-9.1* from the UAE. The searches included combinations of the terms “*mcr-9*,” “*mcr-9.1*,” “colistin resistance,” “United Arab Emirates,” “UAE,” “Enterobacterales,” and “*Salmonella*.” In addition, public genomic repositories, including NCBI GenBank/Pathogen Detection and EnteroBase, were checked for UAE-associated records carrying *mcr-9*/*mcr-9.1*. No prior UAE-associated report or publicly available UAE sequence carrying *mcr-9*/*mcr-9.1* was identified at the time of our search (25th February 2026).

### Antimicrobial susceptibility testing (AST)

The phenotypic resistance of *Salmonella* F9 and F32 isolates was determined using the broth microdilution method with Sensititre™ EUVSEC3 plates (Thermo Fisher Scientific, UK), following the manufacturer's instructions. These plates comprise a panel of 14 antimicrobials representing major therapeutic classes such as β-lactams, aminoglycosides, quinolones, tetracyclines, sulfonamides, phenicols, and polymyxins (including colistin). Interpretation of minimum inhibitory concentrations (MICs) followed the European Committee on Antimicrobial Susceptibility Testing (EUCAST, version 13.0) epidemiological cut-off values (ECOFFs), rather than clinical breakpoints, for *Salmonella enterica* (23). According to these criteria, isolates with a colistin MIC > 2 mg/L were classified as colistin-resistant/non-wild type (suggesting the presence of acquired resistance mechanism) (23). Quality control for antimicrobial susceptibility testing was performed using *Escherichia coli* ATCC 25922 as the reference strain. QC testing was performed alongside each batch of isolates tested on Sensititre™ EUVSEC3 plates.

## Results

The results presented here primarily focus on the genomic characterization, comparative genomic analysis, and contextualization of the *mcr-9.1* resistance determinant. The prevalence data are included only to provide contextual background. Full details of the baseline retail egg survey and comprehensive epidemiological analyses are presented in a separate dedicated publication (17).

### *Salmonella* prevalence and antimicrobial resistance in retail eggs

Of the 380 retail table egg tested samples, 37 samples were positive for *Salmonella* (prevalence: 9.7%, 95% Confidence Interval (CI) 7.2%−13.1%) (Table 1). All *Salmonella* isolates were recovered exclusively from eggshells; no *Salmonella* was detected in egg contents. Two (5.4%) of the *Salmonella*-positive isolates belonged to *S*. Indiana serovar and carried the *mcr-9.1* gene (Table 1). The two *S*. Indiana isolates, designated F-9 and F-32, were recovered from locally produced egg samples collected in November 2024 (Dubai) and January 2025 (Al Ain), respectively (Table 1). *S*. Indiana F-32 isolates differed by only 6 single-nucleotide polymorphisms (SNPs) at genome-level from the earlier isolated *S*. Indiana F-9. *In silico* multi-locus sequence typing identified both F-9 and F-32 as sequence type ST17 (Table 1). In both isolates, phenotypic colistin resistance was not observed despite the presence of *mcr-9.1*; the MIC for colistin was ≤ 1 mg/L for each isolate, within the wild-type susceptible range (Table 1).

Aside from colistin, both *mcr-9.1*-bearing *S*. Indiana isolates exhibited a multidrug-resistant phenotype (Table 1). Isolate F-9 showed resistance to tetracycline (MIC >32 mg/L) and trimethoprim-sulfamethoxazole (MIC >512 mg/L), as well as reduced susceptibility to gentamicin (MIC 8 mg/L) and ciprofloxacin (MIC 0.12 mg/L) (Table 1). Isolate F-32 demonstrated a similar profile, with resistance to tetracycline (MIC >32 mg/L) and trimethoprim-sulfamethoxazole (MIC >512 mg/L), gentamicin MIC of 4 mg/L, and a higher ciprofloxacin MIC of 0.25 mg/L (Table 1). Notably, F-32 also exhibited nalidixic acid resistance (MIC 16 mg/L), which was not observed in F-9 (Table 1). These results indicate that both *S*. Indiana isolates were resistant to multiple antibiotic classes, although neither expressed phenotypic resistance to colistin *in vitro*.

### Genomic features of *mcr-9.1*-positive *S*. Indiana

WGS confirmed that *mcr-9.1*-positive *S*. Indiana isolates shared genomic profiles in their complement of antimicrobial resistance genes. Both carried a suite of nine acquired resistance genes, including the plasmid-mediated colistin resistance gene *mcr-9.1* as well as genes conferring resistance to aminoglycosides (*aadA2, ant(2”)-Ia, aph(3')-Ia*), sulfonamides (*sul1*), tetracycline (*tet(D)*), and quinolone (*qnrA1*) (Table 1). Additionally, both isolates harbored the multidrug efflux pump genes *mdsA* and *mdsB* (Table 1). The only notable difference in the resistome was the presence of the quaternary ammonium compound resistance genes; while both isolates carried the disinfectant resistance gene *qacE*Δ*1*, the gene *qacE* was only detected in F-9.

Beyond antibiotic resistance determinants, the two *S*. Indiana genomes encoded multiple stress tolerance and metal resistance genes (Table 1). Both contained loci for heavy metal resistance, including the *mer* (mercury resistance) operon genes *merR, merT, merA, merD, merE* and other determinants such as *arsC* (arsenic resistance) and *pcoS* (copper/silver resistance) (Table 1). Each isolate also possessed the regulatory genes *golS* and *golT*, which are associated with gold/copper tolerance (Table 1).

**Figure 1:**
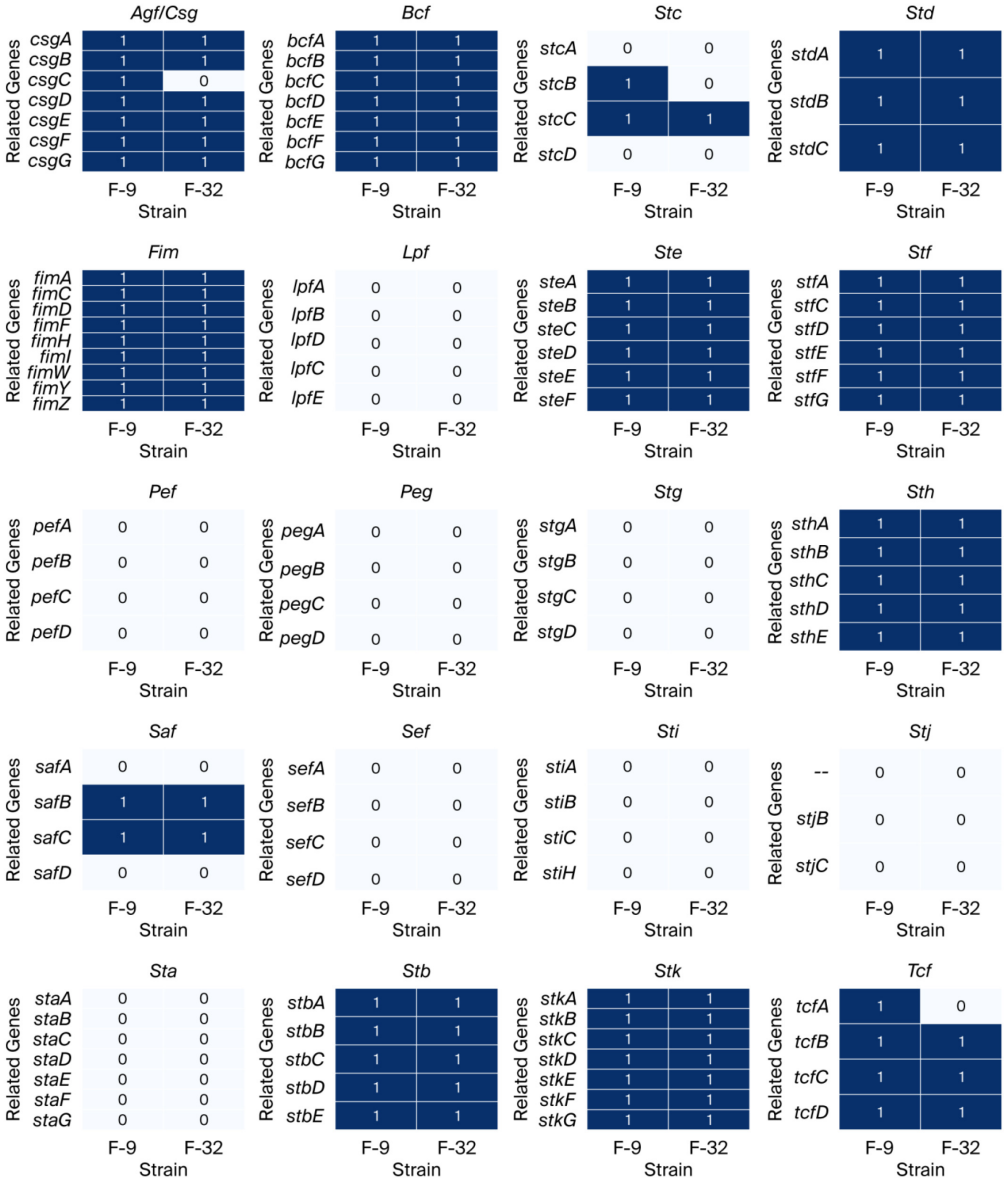
Comparative presence (1; dark blue)–absence (0; light blue) profiling of virulence factors and related genes associated with various fimbrial adherence determinants in the *mcr-9.1* bearing *Salmonella* Indiana F-9 and F-32.

**Figure 2:**
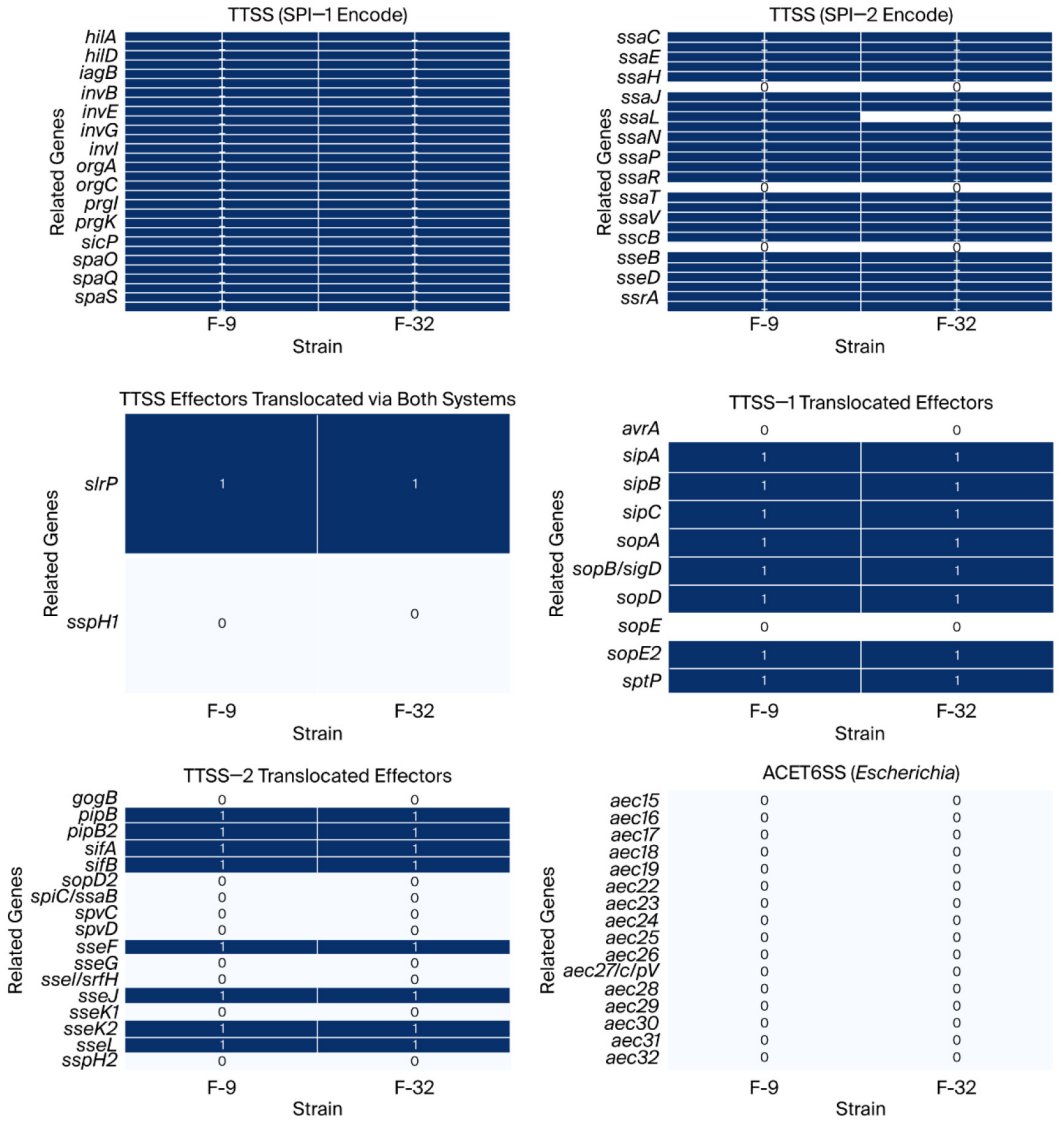
Comparative presence (1; dark blue)–absence (0; light blue) profiling of virulence factors and related genes associated with secretion systems in in the *mcr-9.1* bearing *Salmonella* Indiana F-9 and F-32: Type III Secretion System (T3SS) encoded on *Salmonella* Pathogenicity Island 1 (SPI-1); Type III Secretion System encoded on *Salmonella* Pathogenicity Island 2 (SPI-2); T3SS effector genes translocated via both systems; T3SS-1–translocated effector genes; T3SS-2–translocated effector genes; and Type VI Secretion System (T6SS), ACE cluster (as annotated for *Escherichia***)**.

Overall, F-9 and F-32 *S*. Indiana isolates harbored a sum of 147 and 142 genes across eight virulence factor classes. Virulence factor profiling of the two isolates revealed largely comparable virulence gene portfolios, as shown in Figure 1, where both F-9 and F-32 displayed almost the same pattern of presence/absence for a range of fimbrial adhesion-related genes. Similarly, Figure 2. illustrates that genes associated with Type III secretion systems (SPI-1 and SPI-2) and Type VI secretion systems were uniformly present in both isolates. No major differences in virulence gene content were observed between the two *S*. Indiana isolates.

### Plasmid profiles and localization of *mcr-9.1*

Plasmid analysis indicated that both isolates carried a large ~63 kb plasmid that harbored multiple resistance genes, but the *mcr-9.1* gene was not carried on such plasmid (Table 1). This plasmid was classified by MOB-typing as belonging to cluster AA400 and was predicted to be non-mobilizable (lacking conjugative transfer machinery) (Table 1). Importantly, this plasmid carried the same array of antibiotic resistance genes identified in the whole-genome analysis, including *aadA2, ant(2”)-Ia, aph(3')-Ia, qnrA1, sul1*, and *tet(D)* (Table 1). The difference in *qacE* gene presence between the two strains was also reflected in their plasmid content; the plasmid from F-9 contained the *qacE* gene, whereas the plasmid from F-32 did not (Table 1).

Using a hybrid assembly approach (Unicycler) that combined Illumina short reads with Nanopore long reads, we identified the colistin resistance gene *mcr-9.1* on a large contig (~388, 222 bp) of the draft genome. This contig was assembled as linear (non-circular) by Unicycler, and plasmid profiling with MOB-suite detected no plasmid-associated replicon sequence on it. These findings indicate that in *S*. Indiana strains from table eggs characterized in this study, *mcr-9.1* is likely to be integrated into a non-replicon-bearing large contig likely associated with the chromosome. Notably, the gene was recovered with full coverage and 100% sequence identity to the reference sequence (NCBI accession NZ_NAAN01000063.1; position 364, 689–366, 308). Additionally, a mobile genetic element was observed in the vicinity of *mcr-9.1*. An insertion sequence element, *ISSen1* of the *IS3* family, was identified on the same contig with 99.5% sequence identity to its reference in the *IS*finder database. The presence of this insertion sequence suggests potential for mobility of the *mcr-9.1* region, meaning that while *mcr-9.1* might be putatively chromosomally encoded in the isolates characterized in this study, it resides in a segment that could be prone to transfer or rearrangement under certain conditions.

### Phylogenetic relatedness to global isolates

**Figure 3:**
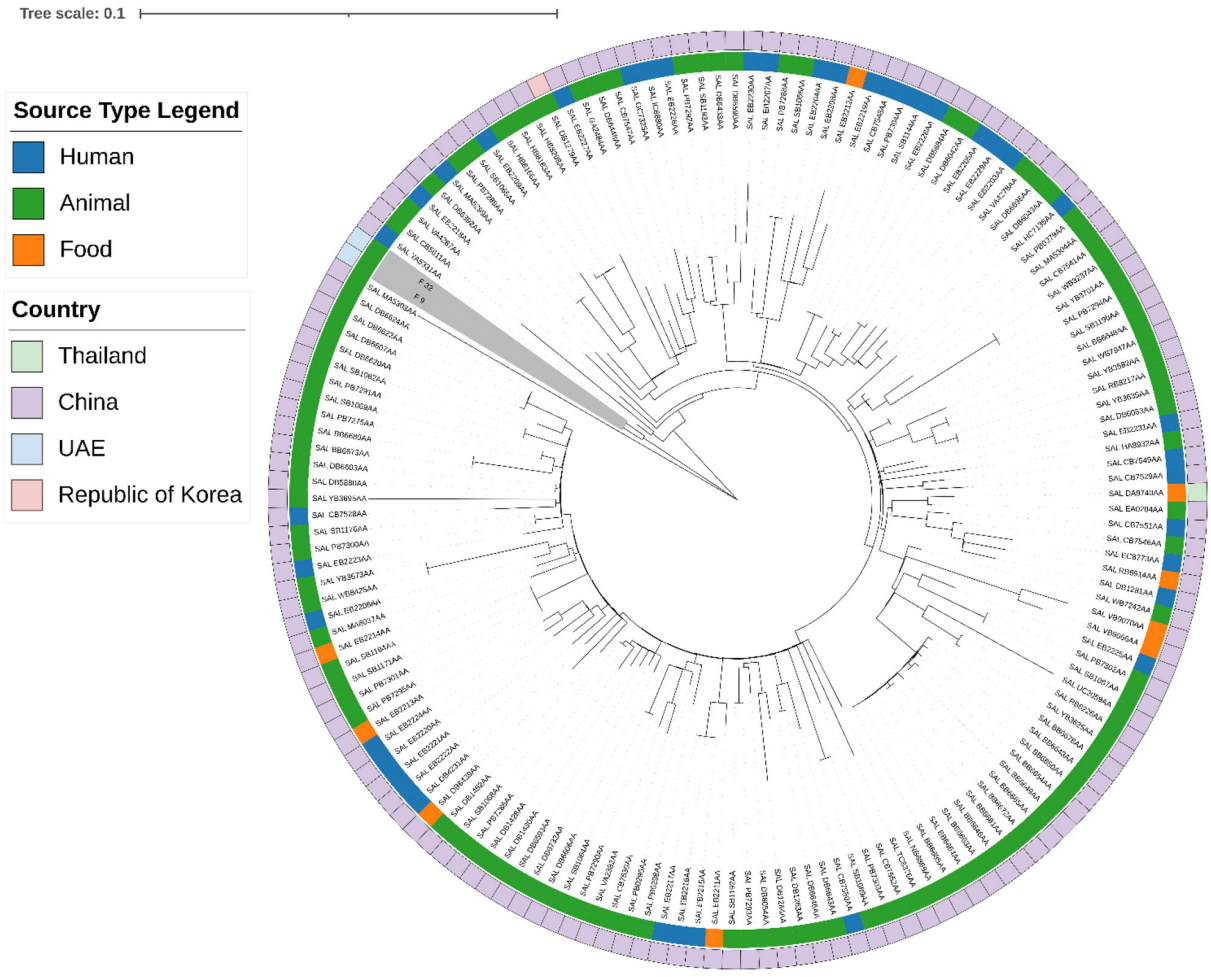
Phylogenetic tree obtained with kSNP4 based on the core single-nucleotide polymorphism (SNPs) of 155 *Salmonella* Indiana ST17 genomes from the EnteroBase database, reported from Asia. The two isolates (F-9 and F-32) identified in this study are highlighted in grey.

A core genome SNP phylogenetic analysis was performed to contextualize the two *S*. Indiana ST17 isolates among international strains. Figure 3 shows a phylogenetic tree of 155 *S*. Indiana ST17 genomes reported from Asia, with isolates color-coded by source and country. In this analysis, the two UAE isolates F-9 and F-32 clustered closely together within a clade predominantly composed of poultry/food-origin isolates (Figure 3). The pair differed by only six core SNPs from each other, indicating a very tight genetic relatedness. The closest phylogenetic neighbor to the UAE *S*. Indiana ST17 isolates differed by 56 SNPs and corresponded to a human-derived isolate from China (2004), followed by closely related isolates spanning wild animal, poultry, and food sources from China collected between 2010 and 2014, with pairwise SNP distances ranging from 63 to 69 (Supplementary Table S1). The study isolates (F-9 and F32) differed by a maximum of 109 SNPs from the most distantly related isolate in the collection. The maximum scored report within the collection was about 150 SNPs with the average core-genome SNP obtained was 75 (Figure 3). The positioning of F-9 and F-32 among mostly animal-derived Asian isolates suggests that these UAE strains share a common lineage with previously reported *S*. Indiana from the region. Notably, no identical genome was present in the international collection, but the close clustering of the UAE isolates with others from Asia points to possible geographic linkages or a similar reservoir for this ST17 lineage (Figure 3).

### The genetic environment of *mcr-9.1*

**Figure 4:**
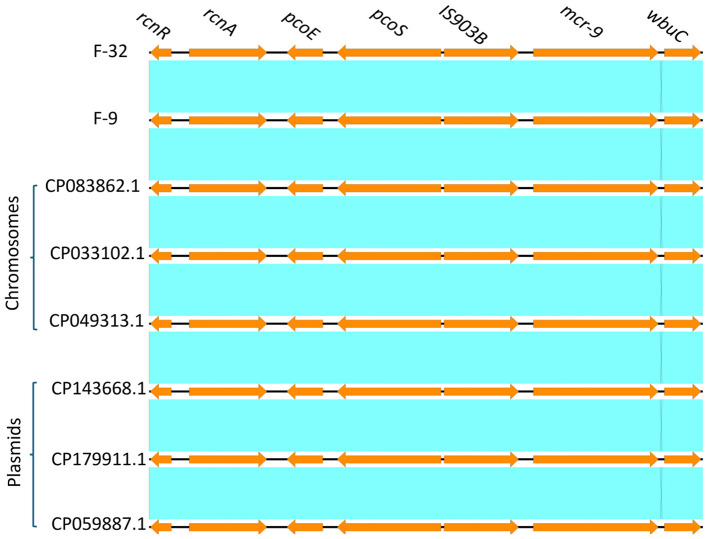
Schematic representation of the genetic environments flanking the *mcr-9.1* locus across each genomic backbone identified in *S*. Indiana isolates (F-9 and F-32), together with closely related reference sequences (100% coverage and 99.9% nucleotide identity) derived from both plasmid and chromosomal contexts.

Comparative genomic analysis revealed that the *mcr-9.1* gene in *S*. Indiana isolates F-32 and F-9 is embedded within a highly conserved genetic environment (Figure 4). In both isolates, *mcr-9.1* was flanked upstream by genes associated with metal resistance and regulation, including *rcnR, rcnA, pcoE*, and *pcoS*, and downstream by *wbuC*, with the insertion sequence IS*903B* consistently located immediately upstream of *mcr-9*. Easyfig alignment revealed 100% coverage and 99,9 % identity between the *mcr-9.1* regions of F-32 and F-9 and multiple reference sequences derived from both plasmid and chromosomal contexts (Figure 4). These included plasmid sequences from *Enterobacter hormaechei* subsp. *xiangfangensis* strain ECC-239 (pECC-239-1; CP143668.1 (human (wound)-China)) and *Salmonella enterica* serovar Strathcona strain N22-0456 (pN22-0456-A; CP179911.1 (human (source was not identified)-Switzerland)), as well as chromosomal sequences from *Enterobacter kobei* strain 11743-yvys (CP083862.1 (human (sputum)-China)), *Enterobacter hormaechei* strain L51 (CP033102.1 (human (Crohn disease)-China)), *Salmonella enterica* subsp. *diarizonae* serovar b,50:-:- (CP059887.1 (human (urinary tract infection)-China)), and *Salmonella enterica* serovar Heidelberg strain CVM N16S321 (CP049313.1 (ground turkey-USA)) (Figure 4). The conservation of gene order, orientation, and nucleotide identity across these diverse chromosomal and plasmid backgrounds indicates the presence of a stable *mcr-9.1* genetic platform.

## Discussion

Veterinary public health research is pivotal toward better understanding of the epidemiology of foodborne AMR along the farm-to-fork continuum. This study provides evidence that retail eggs in the UAE are an exposure node for multidrug-resistant (MDR) *Salmonella enterica*, and that the colistin resistance gene *mcr-9.1* is putatively chromosomally located in two closely related *S*. Indiana ST17 isolates recovered from the retail food chain. From a food safety perspective, these findings are significant because table eggs represent a frequent vehicle, worldwide, for *Salmonella* transmission across farm-to-retail chains (24), while *mcr* genes may remain silent yet inducible under certain conditions (25, 26). Furthermore, as revealed from the study findings, the coexistence of disinfectant and metal tolerance determinants, efflux systems, and multidrug resistance genes in these food-origin isolates highlights the influence of environmental co-selection pressures that operate throughout production, processing, and retail continuum (27, 28).

As far as we could determine from the available literature and public databases, this is the first report of *mcr-9.1* in the UAE. Nevertheless, both *S*. Indiana isolates were colistin-susceptible *in vitro*, consistent with reports that *mcr-9* frequently does not confer increased colistin MICs unless the locus is induced and/or embedded within specific regulatory contexts (e.g., proximity to the two-component system *qseB/qseC*) (9). However, mechanistic studies have shown that *mcr-9* can be inducible and that the presence or expression of adjacent regulatory modules conditions phenotypic resistance (26). Thus, relying on MIC alone can underestimate dissemination potential when inducible expression is possible. It is also worth noting that our hybrid genome assembly efforts pointed the presence of an *IS3*-family insertion sequence (*ISSen1*) nearby the *mcr-9.1* gene characterized in the present study isolates, suggesting potential for mobilization or genomic rearrangement under selective conditions. These observations are consistent with published evidence that, although *mcr* genes (including *mcr-*9) are often plasmid-borne, they can be integrated into chromosomes across taxa where vertical stability is higher but local mobile elements can still catalyze movement (29, 30). Putative chromosomal integration may limit immediate plasmid-mediated transfer, but it also creates a stable reservoir that can persist even as plasmid burdens fluctuate; the nearby *IS* element raises the possibility of future excision/capture by mobile platforms.

Aside from colistin, both *mcr-9.1*-positive *S*. Indiana characterized in this study expressed MDR consistent with their resistomes. Added to that, both isolates harbored disinfectant (quaternary ammonium compounds, QACs) tolerance genes. Recent experimental data in *Salmonella* demonstrate that class 1 integrons with *qacE*Δ*1* can enhance tolerance to benzalkonium chloride and co-associate with MDR, supporting a co-selection paradigm in processing environments that use QACs (31). The genomes also encoded *mer* (merR/T/A/D/E), *arsC*, and *pcoS*, and the *golS/golT* regulators implicated in metal tolerance. Growing evidence links metals (e.g., copper supplementation in feed, residues in litter/manure) with the maintenance/selection of antibiotic-resistance determinants via co-resistance and co-regulation (27, 28). The *mdsAB* efflux module (widely distributed across *Salmonella* serovars and is not specific to *S*. Indiana) adds a further axis of tolerance that may support survival under sanitizers and other stresses, complementing AMR selection pressures along the farm-to-retail continuum (32). Altogether, the *qacE/qacE*Δ*1–mer/ars/pco–mdsAB* constellation identified in these egg-origin *Salmonella* isolates suggests environmental co-selection pressures operating within production and handling environments, thereby highlighting a potential core pathway of concern within the One Health framework. Building on this genomic context, the two *S*. Indiana isolates exhibited highly comparable virulence portfolios, encompassing canonical SPI-1/2 type III secretion systems, type VI secretion systems, and a largely overlapping set of fimbrial adhesion genes. Although virulence phenotypes were not assessed experimentally, the conserved presence of these loci indicates a maintained enteropathogenic potential consistent with foodborne transmission and human infection risk (33, 34).

To evaluate phylogeographic inference of the present study isolates, we constructed a core-SNP context with 155 Asian ST17 genomes. This analysis pointed out that our *mcr-9.1*-positive *S*. Indiana isolates from the UAE retail eggs are well placed inside a predominantly poultry/food clade. This is consistent with phylogenomic work showing ST17's emergence and MDR consolidation in Asia, with strong associations with poultry and food isolates (11, 12). Although phylogenetic analyses in this study place the UAE isolate closest to strains reported from China, including a human isolate and multiple isolates from poultry, food, and wildlife sources, the observed nearest-neighbor distance of 56 SNPs reflects shared lineage background. This relatedness should not be interpreted as evidence of recent transmission, direct importation, or a defined reservoir. Rather, it places the UAE isolates within a broader ST17 genomic background in which related strains have been reported from Asia. Because only two *mcr-9.1*-positive isolates were detected in our egg survey in the UAE and given that some entries on the public genome metadata remain incomplete, any suggestion of cross-border movement, trade-associated introduction, or shared reservoirs should be considered hypothesis-generating and will require confirmation through larger, metadata-rich comparative genomic studies.

Analysis of the genetic environment of *mcr-9.1* in the present study isolates (Figure 4) revealed widespread conservation across different species and genomic locations; this affirms the genetic plasticity of the *mcr-9* locus and supports its potential for dissemination through mobile genetic elements rather than confinement to a single plasmid lineage (4). The consistent association of *mcr-9* with the insertion sequence IS*903B* observed in this study agrees with previous reports describing IS*903B* as a frequent genetic neighbor of *mcr-9* in Enterobacterales (35, 36). IS*903B* has been proposed to facilitate the mobilization and genomic rearrangement of adjacent resistance genes, and its repeated detection upstream of *mcr-9* across diverse bacterial hosts suggests a role in the dissemination of this determinant (35, 36). Notably, in the present study, *mcr-9* was not located on a clearly defined plasmid replicon, supporting previous observations that putative chromosomal integration or association with non-conserved mobile elements may represent an important reservoir for this gene (9). Collectively, these findings suggest that IS*903B*-associated *mcr-9* loci may function as latent reservoirs of colistin resistance with the potential for future mobilization or activation.

## Conclusion

The detection of putatively chromosomally encoded *mcr-9.1* in MDR *S*. Indiana ST17 from retail eggs, alongside fluoroquinolone, tetracycline, and trimethoprim-sulfamethoxazole resistance, underscores a policy-relevant One Health signal at the food interface. Even without current phenotypic colistin resistance, the inducibility of *mcr-9* and the presence of nearby IS elements argue for vigilant genomic surveillance, prudent antimicrobial/biocide/metal use, and supply-chain traceability to interrupt dissemination across human–animal–environment systems. Additionally, the close genetic relatedness of UAE isolates to Asian poultry-origin *S*. Indiana suggests potential import or shared reservoirs, highlighting the need for traceability and cross-border collaboration in AMR mitigation strategies. Overall, our results call for integrated, farm-to-retail surveillance that routinely combines culture and phenotypic screening of resistance, combined with WGS to reveal gene-context localization (chromosome vs. plasmid) and plasmid mobility typing. This aligns with Quadripartite One Health contextual framework to strengthen cross-sectoral AMR surveillance, and with proposals to embed genomics at human–animal–environment interfaces. For eggs specifically, risk communication to consumers and food handlers should reflect the persistent prominence of eggs/egg products in *Salmonella* outbreaks while not presuming traditional serovars (e.g. *S*. Enteritidis) as the only hazard.

## Data Availability

The datasets presented in this study can be found in online repositories. The names of the repository/repositories and accession number(s) can be found below: https://www.ncbi.nlm.nih.gov/, BioSamples SAMN50985254 (F9) and SAMN50985277 (F32).
